# Bistable front dynamics in a contractile medium: Travelling wave fronts and cortical advection define stable zones of RhoA signaling at epithelial adherens junctions

**DOI:** 10.1371/journal.pcbi.1005411

**Published:** 2017-03-08

**Authors:** Rashmi Priya, Guillermo A. Gomez, Srikanth Budnar, Bipul R. Acharya, Andras Czirok, Alpha S. Yap, Zoltan Neufeld

**Affiliations:** 1 Institute for Molecular Bioscience, Division of Cell Biology and Molecular Medicine, The University of Queensland, St. Lucia, Brisbane, Queensland, Australia; 2 Department of Anatomy and Cell Biology, University of Kansas Medical Center, Kansas City, Kansas, United States of America; 3 School of Mathematics and Physics, The University of Queensland, St. Lucia, Brisbane, Queensland, Australia; National Institutes of Health, UNITED STATES

## Abstract

Mechanical coherence of cell layers is essential for epithelia to function as tissue barriers and to control active tissue dynamics during morphogenesis. RhoA signaling at adherens junctions plays a key role in this process by coupling cadherin-based cell-cell adhesion together with actomyosin contractility. Here we propose and analyze a mathematical model representing core interactions involved in the spatial localization of junctional RhoA signaling. We demonstrate how the interplay between biochemical signaling through positive feedback, combined with diffusion on the cell membrane and mechanical forces generated in the cortex, can determine the spatial distribution of RhoA signaling at cell-cell junctions. This dynamical mechanism relies on the balance between a propagating bistable signal that is opposed by an advective flow generated by an actomyosin stress gradient. Experimental observations on the behavior of the system when contractility is inhibited are in qualitative agreement with the predictions of the model.

## Author summary

Mathematical models play a key role in uncovering mechanisms responsible for the formation of patterns in cells and tissues. The well known Turing mechanism based on nonlinear reaction kinetics and differential diffusion explains the formation of static patterns, while positive feedback interactions can generate dynamical structures such as propagating fronts and excitable pulses. Recent studies have demonstrated the importance of mechanical forces that can lead to novel mechanisms of pattern formation such as clustering and oscillations in contractile systems. Here we investigate how contractile forces in mechanically active media can affect bistable front propagation. We found that contraction regulates the front speed or can fully suppress its propagation in space to create a static localized zone. The proposed model provides a new mechanism for cross-talk between mechanical activity of cells and biochemical signaling.

## Introduction

Spatial and temporal patterns of intracellular signaling are thought to play important roles in determining their functional outcomes. This is exemplified by the RhoA GTPase, a major regulator of actomyosin-based contractility in eukaryotic cells [1, 2]. Characteristically, localized RhoA activity defines where contractility is generated and, accordingly, contractile events are distinguished by distinctive subcellular patterns of RhoA signaling. For example, RhoA signaling concentrates at the contractile ring during eukaryotic cell division, co-localizing with the contractile ring that mediates cytokinesis [3]. Another distinctive example occurs in confluent epithelia during interphase: here a prominent zone of active RhoA is found at the apical zonula adherens (ZA) where E-cadherin adhesion couples to actomyosin to generate a zone of high junctional tension [4–6]. As RhoA is necessary for the biogenesis of contractile actomyosin at the ZA [4], this further supports the concept that control of the subcellular expression of RhoA signaling plays a fundamental role in determining where contractility is established within cells. In the present study, we therefore chose the ZA as a model to understand how the spatial expression of RhoA signaling is determined within cells.

The activity of RhoA is controlled by upstream regulators, notably guanine nucleotide exchange factors (GEFs) that activate RhoA by GTP-loading and GTPase-activating proteins (GAPs) that facilitate its inactivation [7–9]. The location of active GEFs is commonly thought to play a key role in defining where RhoA signaling is initiated [2]. For epithelial junctions, we earlier identified the Ect2 GEF as responsible for activating junctional RhoA [4]. As Ect2 itself localized to the ZA, it could be interpreted as a point source for RhoA activation, which ultimately promoted junctional contractility by recruiting and activating non-muscle myosin IIA (NMIIA) [4, 10], an actin-dependent motor protein that is the major contractile force generator in eukaryotic cells. More recently, we also described a feedback network that allows junctional NMIIA to support RhoA signaling, once it has been activated [6]. This feedback involves the scaffolding of Rho kinase (ROCK) by stabilized NMIIA at the ZA, which then antagonizes the junctional recruitment of the RhoA inactivator, p190B RhoGAP, to thereby sustain active RhoA. By combining computational modeling with experimental analysis we found that this biochemical feedback network displayed properties of a bistable system [11], which could account for the stable intensity of signaling that is observed within the RhoA zone of the ZA [6].

**Fig 1:**
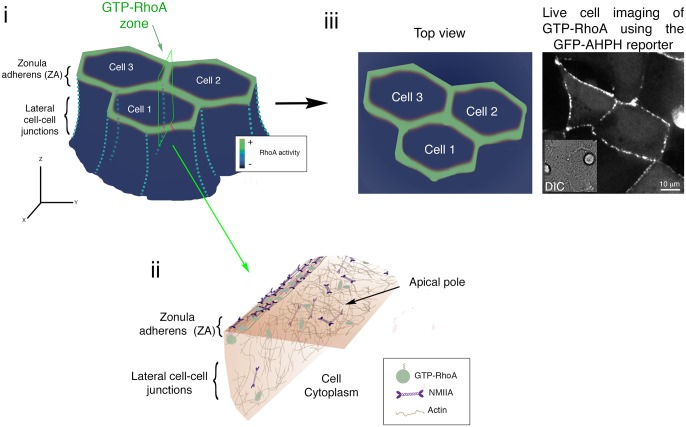
The distribution of RhoA and actomyosin at the epithelial zonula adherens. i. Scheme of coherent epithelial cells and the distribution of the active RhoA zone (green) at the apical adherens junctions (zonula adherens, ZA). ii. Diagram of the actomyosin cortex located on the cytoplasmic side of a cell. The ZA corresponds to a cortical zone enriched in NMIIA and RhoA. Note that RhoA is activated at the ZA, but this lipid-anchored molecule can potentially diffuse horizontally onto the apical surface of the cells as well as vertically down into the lateral cell-cell junctions. iii. Scheme of the top view of the RhoA zone from a group of cells (left) and its comparison with a spinning disk confocal image taken at the apical region of the cells expressing the GTP-RhoA reporter, GFP-AHPH. The inset in the right image corresponds to the same field of view seen by differential interference contrast microscopy (DIC) and shows that the analyzed field belongs to a confluent epithelial monolayer. Note also that the optical (X-Y) plane of the imaging captures the apical surface of the epithelium.

However, RhoA is a lipid-anchored molecule, which can potentially diffuse in the membrane away from its source of activation [12, 13]. Furthermore, mathematical models have revealed that reaction-diffusion systems of membrane-bound proteins can generate dynamic zones that exhibit travelling wave fronts that are not static or confined. In particular, these occur when diffusion is combined with bistability in the underlying dynamical system of non-linear interactions [14, 15], akin to what we identified for the NMIIA-RhoA feedback network of the ZA [6]. Despite this, we observed that the morphology of the RhoA zone at the ZA was stable (Fig 1, S1 Movie), both in its width and the definition of its borders, over time scales (10s min) that are much longer than that of its constituent molecules (RhoA T_1/2_ ~ 0.5 sec) [16]. This observed stability therefore implies that other mechanisms must exist to control the spatial distribution of active RhoA (GTP-RhoA) at the ZA.

Importantly, the NMIIA that influences RhoA signaling at the ZA is part of a contractile cortex that can generate gradients of stress at cell-cell junctions [5, 10]. Recently developed models based on a mesoscopic representation of the actomyosin cortex as an active fluid [17–20] have shown that local differences in contractility can pattern the cortex by generating advective flows that follow the gradients of contractility. We therefore considered whether embedding a bistable signaling network within a contractile medium might influence the patterns of RhoA signaling that can be generated. To test this, we combined the mesoscopic fluid model of the junctional cortex with a simplified scheme where RhoA and NMIIA undergo mutual activation to generate bistability upon dynamic exchange with the cortex. We then compared our model predictions with analysis of the temporal changes of GTP-RhoA in response to perturbations that affect the contractile properties of NMIIA. We conclude that advection and bistability-driven travelling waves exert opposing effects to stably delimit the RhoA zone at the adherens junctions.

## Results

### A minimal model of bistable RhoA-NMIIA signaling coupled to the mechanically active actomyosin cortex

In seeking to understand how meso-scale stability of an NMIIA-RhoA system is achieved at adherens junctions (Fig 1) we considered a minimal model of a RhoA-NMIIA positive feedback loop that can exhibit bi-stability. This corresponds to the case in which RhoA and NMIIA mutually recruit each other to the cell cortex following a Hill type process of cortical adsorption, and dissociate from the cortex with the dissociation rates, *k*_*RhoA*_ and *k*_*NMIIA*,_ respectively [6]:

$$\frac{d\left[RhoA\right]}{dt}=s_{RhoA}\frac{{\left[NMIIA\right]}^{n}}{\kappa_{NMIIA}^{n}+{\left[NMIIA\right]}^{n}}-k_{RhoA}\left[RhoA\right]$$

$$\frac{d\left[NMIIA\right]}{dt}=s_{NMIIA}\frac{{\left[RhoA\right]}^{n}}{\kappa_{RhoA}^{n}+{\left[RhoA\right]}^{n}}-k_{NMIIA}\left[NMIIA\right]$$ (1)

Here, *s*_*RhoA*_ and *s*_*NMIIA*_ are the saturation rates for cortical binding of RhoA and NMIIA, respectively, [*NMIIA*] and [*RhoA*] are the cortical concentration of NMIIA and RhoA, respectively. *κ*_*NMIIA*_ and *κ*_*RhoA*_ are the half-saturation concentrations of NMIIA and RhoA and *n* is the Hill coefficient (*n = 4* for our calculations). Unless otherwise stated we assume *κ*_*RhoA*_ = *κ*_*NMIIA*_. Note also that experimentally, the cortical recruitment of both RhoA and NMIIA is accompanied by their activation [4, 6]; accordingly, we use either RhoA and GTP-RhoA to refer to the same active form of RhoA that is present in the cell cortex.

Then, for simplicity we study the system formed by the cortex as a one-dimensional element, with proteins that are not bound to the cortex located away in the cytoplasm. On the cortex, the two components can diffuse and can also be transported by cortical flow generated when a gradient of active cortical stress is present. The dynamics of the species concentration can be now described in the *x* spatial dimension by the reaction-advection-diffusion equations:

$$\frac{\partial \left[RhoA\right]}{\partial t}+\frac{\partial }{\partial x}\left(v\left(x\right)\left[RhoA\right]\right)=s_{RhoA}\frac{{\left[NMIIA\right]}^{n}}{\kappa_{NMIIA}^{n}+{\left[NMIIA\right]}^{n}}-k_{RhoA}\left[RhoA\right]+D_{RhoA}\frac{\partial^{2}\left[RhoA\right]}{\partial x^{2}}$$

$$\frac{\partial \left[NMIIA\right]}{\partial t}+\frac{\partial }{\partial x}\left(v\left(x\right)\left[NMIIA\right]\right)=s_{NMIIA}\frac{{\left[RhoA\right]}^{n}}{\kappa_{RhoA}^{n}+\left[RhoA^{n}\right]}-k_{NMIIA}\left[NMIIA\right]+D_{NMIIA}\frac{\partial^{2}\left[NMIIA\right]}{\partial x^{2}}$$

where *v*(*x*) is the distribution of the flow velocity, and *D*_*RhoA*_ and *D*_*NMIIA*_ are the cortical diffusion coefficients of RhoA and NMIIA, respectively.

We then consider NMIIA as the mechanically active component of the system, which generates active contractile stress that monotonically increases with its concentration. Assuming that the forces are in quasi-equilibrium, then the drag force is balanced by the stress (*σ*) divergence

$$\gamma v=\frac{\partial \sigma }{\partial x}$$

where *γ* is the friction coefficient. In the above equation, the stress *σ*(*x*) is the sum of the viscous and active stresses [18, 20]

$$\sigma \left(x\right)=\eta \frac{\partial v\left(x\right)}{\partial x}+\varsigma \frac{\left[NMIIA\right]}{\Delta +\left[NMIIA\right]}$$

The viscous stress is proportional to the velocity gradient where *η* is the viscosity coefficient. The active stress is an increasing function of the myosin concentration at the cortex that is linear at small concentrations and when the concentration is much larger than the half-saturation constant Δ it reaches the maximal active stress ς. Without this saturation the positive feedback of myosin on itself can lead to runaway self-contraction and singular solutions in certain regimes [18, 20]. We previously found that this description of actomyosin generated stresses reproduced well the dynamics of actomyosin networks at the lateral adherens junctions of epithelial cells [20].

Introducing non-dimensional variables using the unit length *l =* (*η/γ*)^½^, time unit *τ = l*^*2*^*/D* and unit concentrations *s/k* we obtain

$$\frac{\partial \left[RhoA\right]}{\partial t}+Pe\frac{\partial }{\partial x}\left(v\left[RhoA\right]\right)=\alpha \left(\frac{{\left[NMIIA\right]}^{n}}{\kappa^{n}+{\left[NMIIA\right]}^{n}}-RhoA\right)+\frac{\partial^{2}\left[RhoA\right]}{\partial x^{2}},$$

$$\frac{\partial \left[NMIIA\right]}{\partial t}+Pe\frac{\partial }{\partial x}\left(v\left[NMIIA\right]\right)=\alpha \left(\frac{{\left[RhoA\right]}^{n}}{\kappa^{n}+{\left[RhoA\right]}^{n}}-\left[NMIIA\right]\right)+\frac{\partial^{2}\left[NMIIA\right]}{\partial x^{2}}$$

$$v=\frac{\partial^{2}v}{\partial x^{2}}+\frac{\partial }{\partial x}\left(\frac{\left[NMIIA\right]}{K+\left[NMIIA\right]}\right)$$

The most important non-dimensional parameter of the system is the Peclet number *Pe = ς /*(*γD*) that represents the strength of advection by the contractile flow relative to diffusion (Bois et al, 2011), and *α* = *η/(kγD)* is the ratio of diffusion time vs. the timescale of reaction kinetics. For simplicity we assumed that the parameters of the reaction kinetics and diffusion for *RhoA* and *NMIIA* are the same. Although the real values of these parameters are likely to be somewhat different for the two components [16], this assumption is not essential and does not influence the general qualitative behavior of the system.

### Bistable regime of RhoA-NMIIA signalling

**Fig 2:**
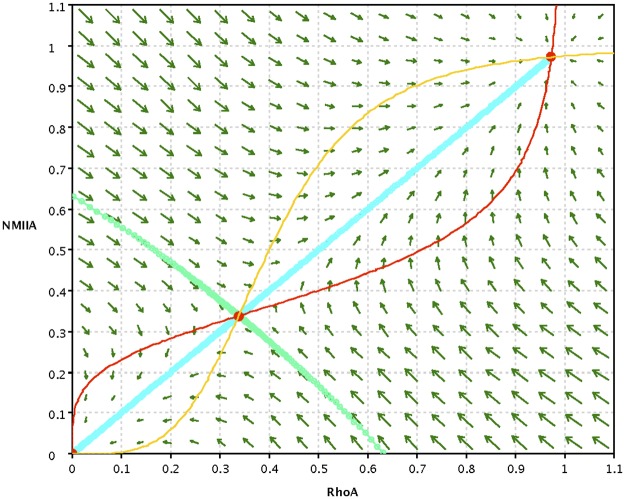
Phase-portrait of the bistable spatially uniform system described by Eq (1). The steady states are at the intersections of the nullclines (red and yellow curves), the light green/blue curves show the stable/unstable manifolds of the unstable steady state, and the arrows show the direction and rate of change at different locations on the *RhoA-NMIIA* phase-plane. The parameters are: *α* = 1, *κ*_*1*_ = *κ*_*2*_ = 0.4, *n* = 4. For this choice of parameters the location of the unstable saddle point is closer to the low fixed point at origin.

To understand the system’s behavior, we first consider the *RhoA-NMIIA* system with the two interacting components uniformly distributed in the cortex without diffusion and advection. An example of the typical phase-portrait and nullclines of this system is shown in Fig 2. The uniform bistable system has two stable steady states. One corresponds to low contractility, where both species are uniformly and poorly recruited to the cortex (i.e. [*RhoA*] = [*NMIIA*] = 0). The second state represents a contractile state, similar to the one found at steady state at the epithelial ZA [6], distinguished by effective recruitment to, and a high uniform concentration of NMIIA and RhoA at, the cortex ([*RhoA*]^*high*^, [*NMIIA*]^*high*^). The system also has an intermediate unstable steady state. The trajectory leading to this saddle point on the phase diagram (stable manifold, green curve in Fig 2) forms the boundary that divides the basins of attraction of the two stable steady states. We note that the bistability is parameter dependent. As an approximate condition, it requires that the half-saturation constants for the activation of each component should not be larger then the corresponding maximum concentrations of the other component. Otherwise there is only one intersection of the nullclines and the stable contractile state does not exist.

### Analysis of the model in the presence of diffusion without advection

**Fig 3:**
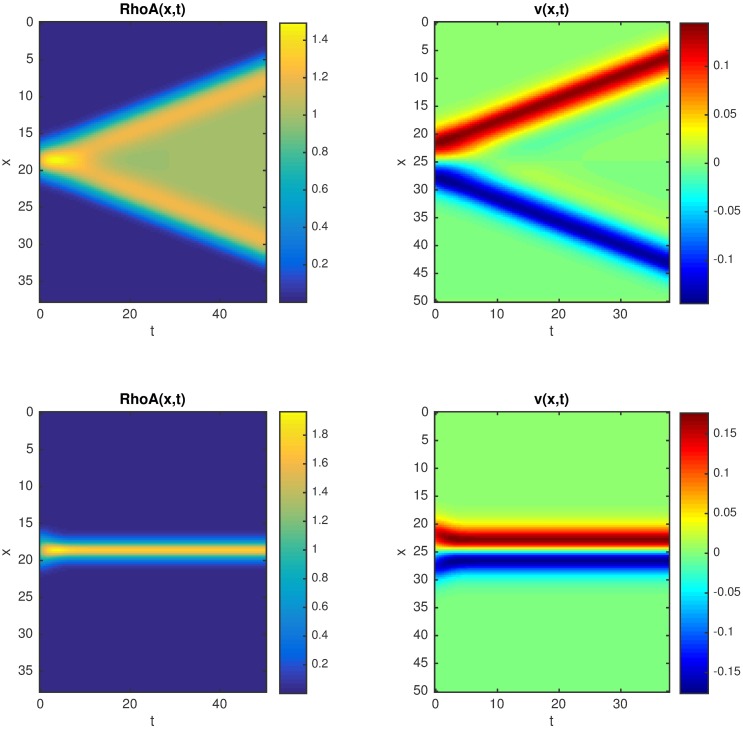
Space-time plots of the concentration field *RhoA(x*,*t)* and flow field *v(x*,*t)*: top row: *Pe* = 10, the front propagates with constant speed; bottom row: *Pe* = 12, stationary *RhoA* zone; the other parameters are: *α* = 1, *κ*_*1*_ = *κ*_*2*_ = 0.2, *K* = 1

In the case of the spatially distributed system in a static medium with diffusion but without contractile stress and flow (*v = 0*), the solution of the reaction-diffusion system produces co-moving cortical travelling fronts for both NMIIA and RhoA (similar to the one shown in the top-left panel of Fig 3). These correspond to asymptotic solutions of the general form *C*(*x*,*t*) = *f*(*x-st*) that connect the two stable steady states, and the constant *s* is the speed of the propagating front [21, 22]. If in a certain part of the domain the system is initially in the “high” state ([*RhoA*]^*high*^, [*NMIIA*]^*high*^) and elsewhere in the low state ([*RhoA*]^*low*^ = [*NMIIA*]^*low*^ = 0), then the boundary between the two regions moves with speed *s*, so that the *dominant* steady state, which is determined by the parameters of the bistable dynamics, expands in space while the other shrinks. We choose the parameters of the system such that the stable steady state with high concentration of NMIIA and RhoA is dominant ([*RhoA*]^*high*^, [*NMIIA*]^*high*^*)* over the low concentration steady state.

We then model the case when a uniform state with ([*RhoA*]^*low*^ = [*NMIIA*]^*low*^ = 0 is perturbed by a spatially localized pulse of RhoA activation, whose amplitude is larger than the threshold determined by the saddle point. This leads to a local increase in [*RhoA*] and [*NMIIA*] followed by the propagation of the “high” state (([*RhoA*]^*high*^, [*NMIIA*]^*high*^) with constant speed in both directions until a uniformly “high” state is reached in the whole domain. Therefore, this model implies that in the absence of advection the locally recruited, active RhoA would tend to spread away from the source (i.e. the ZA) as a consequence of diffusion driven by the action of the bistable signaling. Of note, this spreading would be limited by other conditions: for example, if the total amount of either RhoA or NMIIA is limiting in the system, then the total amount of protein may not be sufficient to propagate the wave beyond a certain distance [23, 24]. The decay rates of RhoA or NMIIA may also vary in different cortical regions, for example having higher values in regions outside the ZA [6].

### Analysis of the model in the presence of NMIIA-driven advection

**Fig 4:**
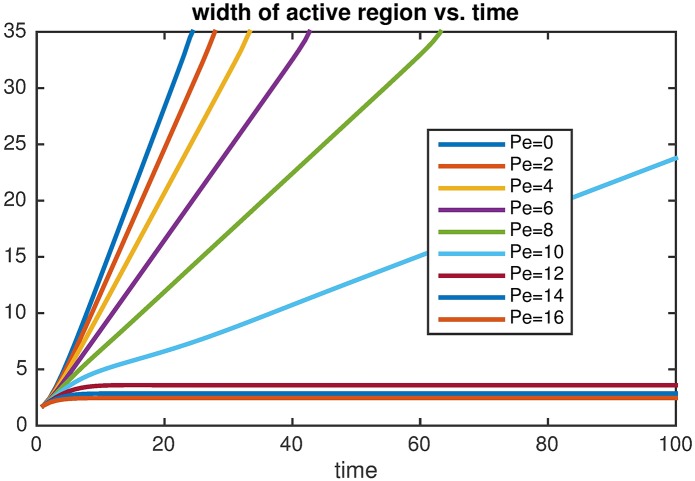
The width of the contractile zone (with higher concentrations of *RhoA* and *NMIIA*), defined as the integral of the *RhoA* concentration over the whole domain, as a function of time, starting from a localized initial condition for a range of different contraction strengths (*Pe = 2* to 16). Note, that i) the slope (i.e. propagation speed) decreases as *Pe* is increased and there is a transition to a stationary contractile zone when *Pe* > 10. (*α* = 1, *κ*_*1*_ = *κ*_*2*_ = 0.2, *K* = 1); ii) the curves corresponding to Pe = 0–10 propagate with constant speed until they reach the end of the computational domain.

Next we investigate the dynamics of the system in the presence of cortical advection generated by myosin activity where both NMIIA and RhoA are advected by the flow. In our model advection is driven by a mechanical stress gradient that is induced by the local recruitment of NMIIA. When the medium is contractile the numerical simulations show two qualitatively different cases (Fig 3). When the contractility is relatively weak, i.e. the Peclet number is lower than a certain threshold, we find again travelling front solutions as in the case of a medium with diffusion but without advection, however the propagation speed is reduced (Figs 3 and 4). The gradient of the active stress generates actomyosin flow regions that are localized around the edge of the moving fronts directed towards the RhoA zone. Increasing the Peclet number causes the propagation speed to decrease gradually, and when *Pe* is above a certain threshold a stationary localized zone of RhoA and NMIIA forms that is sustained by a convergent flow (Figs 3 and 4). Moreover, we found that stationary results are independent of whether we start simulations using either small or big active zones as initial conditions. Thus, the diffusive spread of the molecular components balanced by a steady contractility-driven directed transport maintains a signaling zone that is stable for time scales much longer than the time scales of cortical dissociation and diffusion.

**Fig 5:**
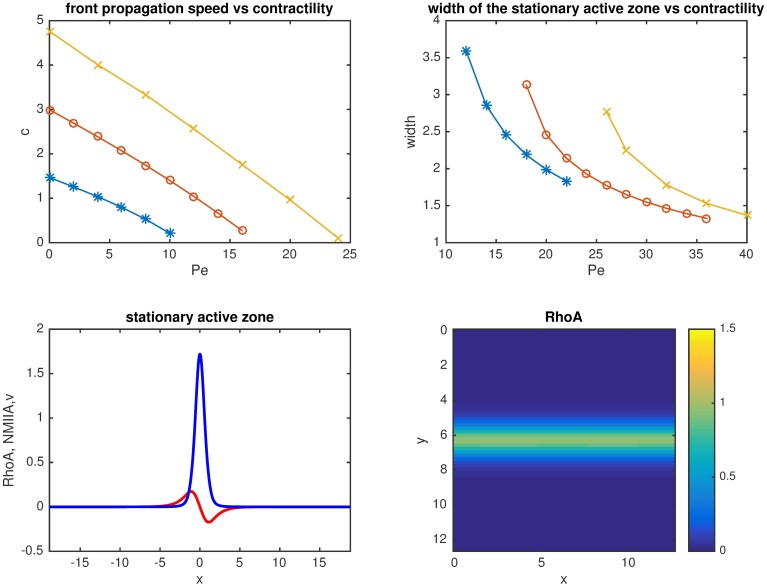
Propagation speed of the front (A), width of the stationary active zone (B) as a function of the Peclet number for α = 1, 4 and 10. (C) Example profile of stationary active zone and the corresponding flow profile (*Pe* = 15, α = 1). (D) Example of a stationary active zone in simulations in two dimensions (*Pe* = 15, α = 1).

We then analyzed the extent to which interaction between diffusion, bistable signaling and NMIIA-dependent advection could influence the stabilization and size (i.e. width) of the RhoA zone. Of note, the RhoA zone of the ZA constitutes a small proportion of the height of the lateral surface of epithelial cells (e.g. <5%). For this we first analyzed the effect of varying the Peclet number with a fixed value of *α* (Fig 5A and 5B). We observed that increasing contractility has a pronounced effect on narrowing the RhoA zone, decreasing it to 1.5 units length for a biologically relevant lower limit of Pe~30 for the lateral junctions where junctional contractility is ~5 times lower than at the ZA [20]. When contractility is further decreased, then the predicted RhoA zone width first increases up to about 3 length units, after which it tends to infinity, which implies that the system cannot generate a stable RhoA zone within the spatial length scales of the lateral cell-cell interface (Fig 5B and 5C). In contrast to the case when contractility was varied, having faster reactions (large *α*) led to wave fronts that propagate faster and therefore require stronger contractile flow (i.e. larger *Pe*) in order to create a stationary active zone (Fig 5A and 5C).

We also tested the prediction of the one-dimensional model in two-dimensional simulations. (For details of the implementation of the two-dimensional simulations see [20]) We found qualitatively similar behavior to the 1D case. Stripe-like initial conditions generate a travelling front that becomes stationary when the Peclet number is sufficiently high. An example of the distribution of the stationary active zone is shown in Fig 5D.

### Analysis of the case in which only NMIIA is advected by flow

In the above model both *RhoA* and *NMIIA* are advected by flow. However, for the contractile cortex, *NMIIA* is likely to be more directly affected by the flow than *RhoA*. *NMIIA* interacts directly with the cortical F-actin network that is advected by contractile flow [25]. In contrast, *RhoA* is thought to principally interact with the lipid bilayer of the plasma membrane through its farnesyl anchor [2]. Accordingly, we extended our numerical analysis to consider the case where only *NMIIA* was advected by flow.

**Fig 6:**
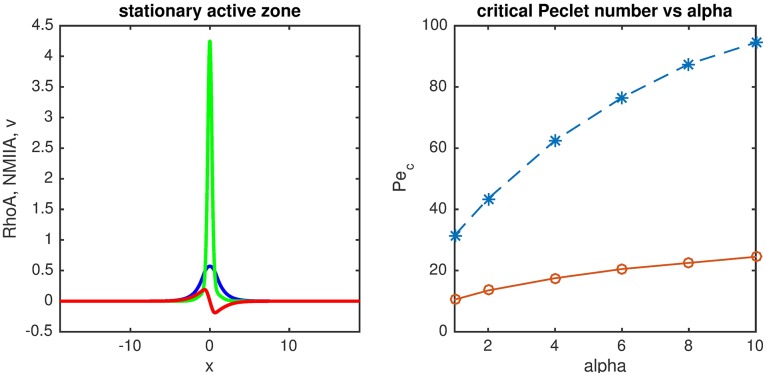
Numerical results for the system in the case when only the force-generating component (*NMIIA)* is transported by the flow. (A) Stationary active zone (*Pe* = 35, *α* = 1). (B) The critical Peclet number (i.e. the value at which Pe trends when c→0, see also Fig 5A) as a function of the strength of the bistable signaling (parameter α), in the case when both components are affected by the flow (continuous line) and when only the contractile component (*NMIIA*) is advected (dashed line).

We found that even in this case the fronts of *RhoA* and *NMIIA* remain co-localised (Fig 6). This is due to the biochemical coupling between the two species, as they mutually contribute to each other’s recruitment from the cytosol mediated by the NMIIA-RhoA feedback loop. Therefore it is sufficient for contractile flow to act on one of the species in order to produce qualitatively similar behavior as described above. However, there is, of course, change in the quantitative details of the effect of contractility (Fig 6). When only one species is advected by the flow, in general there is a weaker reduction of the front speed and the transition to the stationary front regime is also shifted to higher Peclet numbers, i.e. the formation of a stable RhoA zone requires stronger contraction. In addition, when a stationary active zone is generated the distribution of the mechanically active component *NMIIA* is narrower in comparison to the zone formed by *RhoA*, which is not transported by the flow (Fig 6). We also expect that a similar behavior would be observed for the case where the species exhibit different reaction kinetics of binding to the cell cortex, as RhoA has a turnover kinetics of binding to the cortex that is 10 times greater that the observed for NMIIA.

### Analysis of the system in response to inhibition of contractility

**Fig 7:**
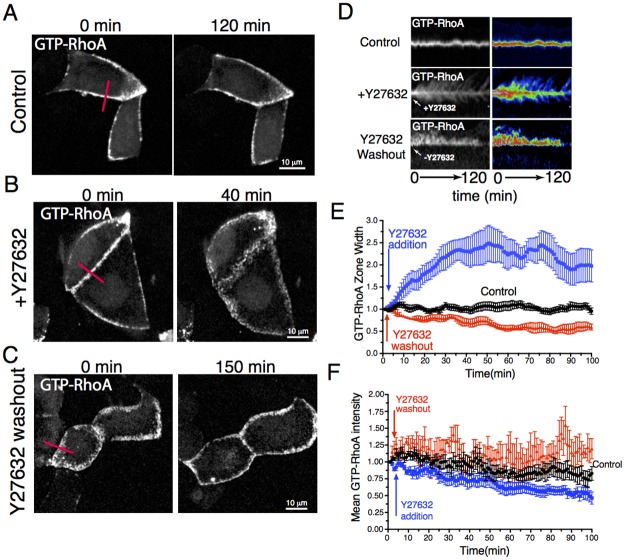
Response of the GTP-RhoA zone at the zonula adherens to changes in actomyosin contractility. Spinning disk time lapse imaging of confluent MCF-7 epithelial cell monolayers expressing the GTP-RhoA reporter GFP-AHPH in control cells (Control, see also S1 Movie); before and after Y-27632 addition (60 μM, time of addition = 3 min, +Y-27632, see also S2 Movie) and before and after Y-27632 washout (S3 Movie). In the last case cells were pre-treated with 60 μM Y-27632 for 1 hour. A-C. Snapshot of the apical region of cells showing the distribution of GTP-RhoA at 0 and 120 min of image acquisition (A), before and after 40 min of Y-27632 treatment (B) and before and 150 min after removal of Y-27632 (C). D. Kymograph analysis of the GTP-RhoA zone for the conditions in A-C. Panels correspond to the analysis of the cell-cell junction indicated in A-C and S1, S2 and S4 Movies. E and F. Quantitation of the temporal changes (normalized to time t = 0) of the width (E) and mean fluorescence intensity (F) of the GTP-RhoA zone at the epithelial zonula adherens. Plots are mean ± SEM from 9 junctions from 3 independent movies.

We then sought to test the predictions of this model for the RhoA zone of the ZA. We imaged active, GTP-loaded RhoA (GTP-RhoA) using a location biosensor derived from the C-terminus of anillin (GFP-AHPH, Fig 7). This reporter binds specifically to GTP-RhoA, and thus its localization identifies the location of GTP-RhoA [6]. As previously described [6], GTP-RhoA localized in a prominent ring-like zone at the apical poles of confluent MCF7 mammary epithelial cells (Figs 1 and 7A). Kymographic analysis of live-cell movies further confirmed that both the spatial definition of the zone (its width and definition of boundaries) and its signal intensity were stable over the 2 hr duration of the movies (Fig 7A and 7D–7F, S1 Movie). Based on our computational analysis we now predicted that diffusion driven by bistable signaling between NMIIA and RhoA would tend to cause an outward-travelling front of GTP-RhoA, unless this was counteracted by local mechanical stress (advective flow) at the ZA.

We tested this prediction qualitatively by monitoring the spatiotemporal response of the GTP-RhoA zone when NMII was inhibited. For technical reasons, we blocked contractility using the ROCK inhibitor, Y-27632, as this drug is compatible with live-cell imaging. Moreover, due to the geometry of cell in the monolayer and the position of the Rho zone (Fig 1), measurements could be only done in the XY plane of the cortex parallel to the plane of the microscope stage, which corresponds to the apical surface of the epithelial monolayer. Whereas control cells (Fig 7A, 7D and 7E), retained GTP-RhoA as a tightly defined band at apical junctions, addition of Y-27632 caused cortical GTP-RhoA to diffuse progressively outwards from the junction, leading to a broader zone within ~50 min of adding the drug (Fig 7B and 7D–7F, S2 Movie). This broadened zone then faded after 2 hrs treatment, likely due to inactivation of GTP-RhoA by the cortical recruitment of p190B RhoGAP [6]. Indeed, when this experiment was performed in p190B RhoGAP RNAi cells, we found that the GTP-RhoA zone persisted and displayed even more pronounced outward flux from the junction after treatment with Y-27632 (S3 Movie).

We then also compared the effect of increasing contractility in cells with the results from simulations. To mimic the effect of increasing contractility we first treated cells with Y-27632 (1 hr) to inhibit NMII, and then washed out the drug to allow contractility to recover. In the experiments shown, imaging began immediately upon drug wash-out (Fig 7C–7F, see also S4 Movie). Kymographs showed that the GTP-RhoA zones were initially broad and comparable to what we observed after adding Y-27632 (Compare Fig 7C, 0 min with Fig 7B, 40 min). After wash-out of the drug, the GTP-RhoA zone became progressively narrower (Fig 7C–7E), associated with a slight increase in the mean fluorescence intensity of GTP-RhoA (Fig 7F).

**Fig 8:**
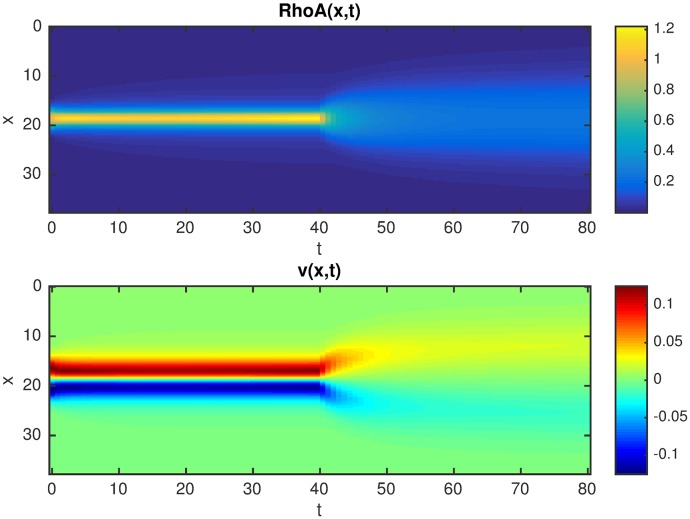
Space-time plot of the response to an external perturbation at *t* = 40, that decreases the stability of *NMIIA* by a 2-fold increase of its decay rate and at the same time the contractility of *NMIIA* is switched off (i.e. *Pe* = 0). The initial parameters are: *Pe* = 8, α = 0.02, κ_1_ = κ_2_ = 0.2, *K* = 1.

We then compared these results with numerical simulations of similar experiments. An example of a simulation that mimics the effect of the inhibitor is shown in Fig 8. Here, after reaching the steady state with a stationary active zone, an external perturbation eliminates contractility and bistability in the system by increasing the decay rate of *NMIIA*, as is predicted to occur experimentally upon addition of Y-27632. We further assume that molecular turnover (degradation of GTP-RhoA) is somewhat slower than diffusion (i.e. small *α*). Numerically, we found that this caused a biphasic change in the RhoA zone: a transient broadening that is followed by overall complete inactivation, that matches qualitatively with what we observed in our experiments.

## Discussion

The stable RhoA zone of the epithelial ZA provides a challenging problem of biological organization. In particular, the RhoA zone in established epithelial monolayers displays clearly defined borders whose width is stably maintained, despite being comprised of molecules that can potentially diffuse in the plasma membrane away from their source of activation. Our current analysis leads us to propose that this paradox is resolved by the opposed action of two processes: bistability-driven spreading and contractile advection. Importantly, both processes derive from the functional interplay between active RhoA and cortical NMIIA. The key lies in the fact that the higher concentration of NMIIA found at the ZA can contribute both to bistable feedback and drive contractile flows. Then, the tendency for bistable feedback to promote the outward propagation of RhoA and NMIIA by travelling wave fronts is countered by inward advection promoted by the gradient of contractility.

Travelling front solutions of reaction-diffusion systems are well known in mathematical models of diverse biological systems [14, 15, 21]. Examples include neuronal action potentials [26], calcium waves (e.g. in cardiac muscle), actin polymerisation waves in motile cells [27], wound healing [28–31], cancer cell invasion [32–34], and invasive species in ecology [35]. This kind of behavior typically arises from the combination of diffusion and bistability of the underlying dynamical system of nonlinear interactions between the components [22]. The bistability identified in the NMIIA-RhoA feedback network is capable of generating such travelling waves in simulations and this was supported experimentally by the observation that the RhoA zone broadened progressively when contractility was inhibited. This is consistent with GTP-RhoA diffusing away from its source at the ZA until it was inactivated by p190B RhoGAP. It further implies that when NMIIA activated by RhoA is capable of diffusion, then the system will tend to generate travelling waves.

However, we further found that when the bistable system occurred in a contractile medium, then the propensity for travelling wave fronts to propagate away from the ZA could be counteracted by advection. Depending on the strength of contractility, this resulted in fronts that propagate slower than in a passive medium or, when the contractility is sufficiently strong, the front may be transformed into a stationary, localized active zone. This is consistent with previous modeling studies that showed that advection by fluid flows can have nontrivial effects on propagation of reaction-diffusion fronts [36, 37] In the present case of a contractile RhoA-NMIIA cortex, directional transport due to the flow produced by the stress gradient at the border of the active zone can counteract the propagation of the front. We found that this phenomenon is robust and persists even when only one component of the coupled dynamical system (NMIIA) is directly affected by the flow.

Our analysis implies that the balance between the stress gradient and bistable wave-front propagation can suffice to determine the size and stability of the RhoA zone. This is consistent with experimental evidence that a contractile gradient exists at cell-cell junctions, where stress is greatest at the ZA, which is predicted to be capable of counteracting the direction of wave front propagation [5]. The ability of cells to maximize stress at the site of the ZA is likely to reflect multiple mechanisms. These include both the localization of the RhoA activator Ect2 [4] that initiates RhoA signaling at the ZA and the ability of bistability to potentiate the magnitude of the RhoA signal and spatially constrain it [6]. However, additional factors can also contribute to support the contractile gradient, including F-actin stabilization by N-WASP at the ZA [5, 38] as well as other signaling pathways, like Akt, which have been shown modulate the width and organization of the zonula adherens [39]. How these additional factors may influence the behaviour of the system that we have characterized will be an interesting issue for future research.

**Fig 9:**
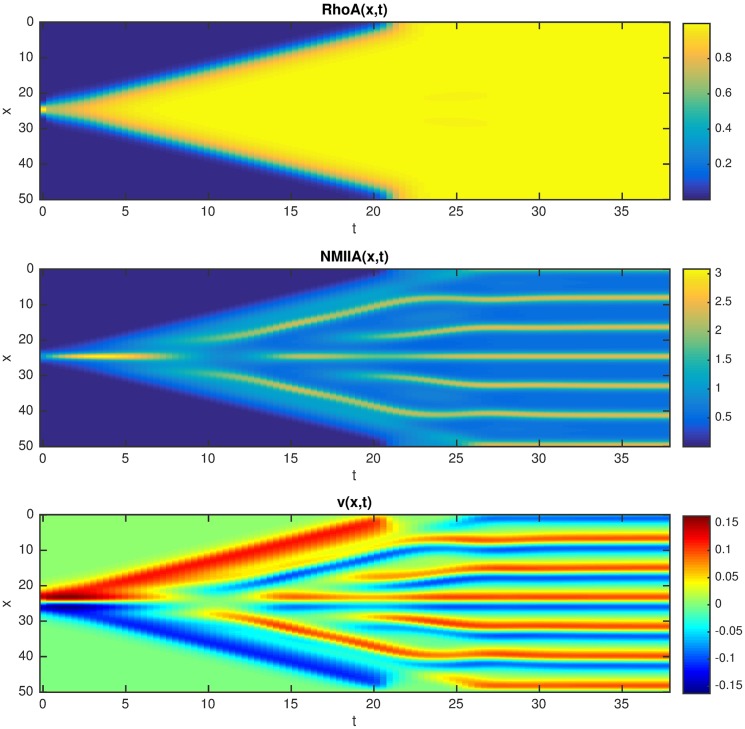
Space-time plots in the case when only the force-generating component (*NMIIA)* is transported by the flow. showing that for intermediate *Pe* there is a clustering instability of the contractile component inside the expanding active zone (*Pe* = 20, *α* = 1).

This general model of bistability within a contractile medium has the capacity to generate diverse spatial patterns, depending on the parameters that are set. For example, the front propagation may coexist with the clustering instability characteristic of active contractile systems without biochemical coupling [18, 20] Indeed, we found computationally that in the propagating front regime the uniform active zone may be subject to clustering, where the contractile component forms a periodic pattern of high concentration clusters behind the front, while the non-advected component remains uniform (Fig 9). Such behavior is restricted to intermediate *Pe* values, since at small *Pe* the contractility is below the clustering instability threshold, while at large *Pe* the front propagation is blocked and the active zone is restricted to a single cluster. Alternatively, there are biological circumstances when junctional RhoA signaling is downregulated to facilitate morphogenesis [40]. Here, maneuvers that broaden the RhoA zone may provide a pathway for junctional mechanics to be altered without altogether abolishing the apical ZA. Ultimately, the capacity for the NMIIA-RhoA to act as a shape-generator reflects the fact that this system couples bistability to a contractile medium. Moreover, the combination of RhoA with NMIIA occurs in many other cellular circumstances, from cell division to locomotion. The basic principles of the system that we have characterized at the ZA are readily applicable to these other situations and, potentially, to other mechanically active contractile reaction-diffusion systems.

## Materials and methods

### Cell culture, transfections and drug treatments

Y-27632 (no. Y0503) was from Sigma. The GFP–AHPH location RhoA biosensor was a kind gift from M. Glotzer (University of Chicago, USA, [41, 42]).

MCF-7 cells were from ATCC and cultured in DMEM; supplemented with 10% foetal bovine serum (FBS), 1% non-essential amino acids, 1% L-glutamine, 100 U/ml penicillin and 100 U/ml streptomycin. For the expression of the GTP-RhoA reporter (GFP-AHPH, [6]) and live cell imaging, cells were grown till 80–90% confluent on glass bottom dishes (Shengyou Biotechnology), transfected using Lipofectamine 3000 and analyzed by live cell spinning disk microscopy. Cells were imaged in clear Hank’s balanced salt solution supplemented with 5% FBS, 10 mM HEPES (pH 7.4) and 5 mM CaCl_2_. Only cells expressing low levels of GFP–AHPH were used for analysis.

p190B RhoGAP was depleted using a combination of two siRNAs designed against the 3’ UTR region of p190B RhoGAP mRNA (NM_001030055.1). Sequences are as follows: siRNA_5007 Sense: GCAUGACUGGAGAGGUUUATT and siRNA_5063 Sense: GCUGCUGCAUGCAACCUUATT).

For live-cell imaging using Y-27632, dishes were mounted on the microscope stage, cells were then selected for imaging and time-lapse imaging (1 Z-stack/minute) was initiated. After three minutes of imaging, 1 volume solution of movie media with 120 μM Y-27632 was added to the cells which gave a final concentration of 60 μM. For these experiments, a higher concentration of Y-27632 (compared to other reports citing this drug usage) was used, to capture its effects on the organization and distribution of the Rho zone in a relatively short time scale. Although similar phenotypes, but with a slow kinetics, were observed when Y-27632 was used at 30 μM.

In Y-27632 washout experiments, cells were pretreated with 30 μM Y-27632 for 1 hour in 2 ml of movie-media. Then 1.7 ml of media was removed and cells were placed on the microscope. After 3 minutes of time lapse imaging (as indicated above), 1.5 mL of fresh imaging media (without Y-27632) was added to the cells and time lapse was continued.

### Spinning disk microscopy and kymograph analysis of the GTP-RhoA signaling zone

Live-cell imaging of GFP–AHPH was performed on a Zeiss inverted spinning-disc confocal microscope equipped with a 63 × 1.3 NA multi-immersion immersion objective (Zeiss), a CSU-X1-A Yokogawa spinning-disc unit and two Roper Evolve EMCCD 512 × 512 cameras. Z-tacks (~30 slices, 512x512 pixels) with a 1 μm z-step where acquired every minute with a spatial resolution of 0.211 μm/pixel.

For kymograph analysis and measurements of fluorescence intensity and width of the Rho zone at the apical adherens junctions, sum projections in the Z-axis where performed on the movies and kymographs of lines traced orthogonal to apical adherens junctions and measurements of the junctional position in it where obtained as described in [43] using a custom made Image J plugin. From this data, the position of the junction in every frame was set as X = 0 while the limits of the zone was obtained by applying a threshold by above the background on the obtained kymograph and analyzed over time. From this then the average fluorescence intensity of GFP-AHPH and width of the Rho zone was measured using a custom made MatLab script.

## Supporting information

S1 Movie Time lapse imaging (maximal projection) of MCF-7 cells expressing the GTP-RhoA reporter GFP-AHPH using spinning disk microscopy. Projections are from stacks (30–40 slices from the whole cell) with a dZ = 0.3 μm. Images were taken every 1 min, Total acquisition time 120 min. See also Fig 6A.
(AVI)

S2 Movie Time lapse imaging of MCF-7 cells expressing GFP-AHPH using spinning disk microscopy. Y-27632 was added to a final concentration of 60 μM (time of addition = 3 min). Images were taken every 1 min, total acquisition time 99 min. See also Fig 6B.
(AVI)

S3 Movie Time lapse imaging of p190B knockdown MCF-7 cells (see Materials and methods) expressing GFP-AHPH using spinning disk microscopy. Y-27632 was added to a final concentration of 60 μM (time of addition = 3 min, total acquisition time 200 min).
(AVI)

S4 Movie Time lapse imaging (maximal projection) of MCF-7 cells expressing GFP-AHPH using spinning disk microscopy. Cells where pretreated with 30 μM Y-27632 for 1 hour, mounted on the microscope stage and after 3 min of image acquisition, the Y-27632 concentration was diluted by 85% by adding fresh media (Y-27632 washout). Imaging (1 stack/minute) continued for the next 150 min. See also Fig 6C.
(AVI)
